# Adaptionen des Spiegeltrainings bei Phantomschmerz mit Teleskop-Phänomen

**DOI:** 10.1007/s00482-023-00745-2

**Published:** 2023-09-04

**Authors:** Marina M. Finnern, Josepha Zimmer, Herta Flor

**Affiliations:** Hochschulambulanz für Psychologische Psychotherapie des Instituts für Neuropsychologie und Klinische Psychologie, Zentralinstitut für Seelische Gesundheit, J5, 68159 Mannheim, Deutschland

**Keywords:** Teleskop-Phänomen, Spiegeltherapie, VR-gestützte Therapie, Amputationsschmerz, Neuropathischer Schmerz, Telescoping phenomenon, Mirror therapy, Virtual reality-assisted therapy, Phantom limb pain, Neuropathic pain

Ein 53-jähriger Patient sucht im September 2020 eigeninitiativ unsere Spezialambulanz für chronische Schmerzen mit dem Wunsch nach Behandlung seiner Schmerzen auf. Er leidet seit 23 Jahren an Phantomschmerz infolge eines Motorradunfalls 1997, bei dem es zum Ausriss des Plexus axillaris und einer subtotalen Amputation des linken Arms im Schultergelenk gekommen ist. Die aktuelle Schmerzintensität bei Aufnahme beträgt 5–6 auf einer numerischen Rating-Skala (NRS) mit den Endpunkten 0 und 10. Gleichzeitig nimmt er seinen linken Unterarm um 9 cm verkürzt wahr (Teleskop-Phänomen). Mehrere stationäre Schmerzbehandlungen mit anschließender ambulanter Weiterbehandlung hatten vorrangig eine medikamentöse Einstellung zum Ziel. Aufgrund des Ausrisses des Plexus axillaris sei es nicht möglich gewesen, eine myoelektrische Prothese zu erhalten. Seine mechanische Prothese erhielt er erst ca. ein halbes Jahr nach dem Unfall und trägt sie aufgrund eines Druckgefühls am Stumpf kaum bis gar nicht. Die Medikation zu Behandlungsbeginn lag bei 30 mg Morphin (1-1-1), Gabapentin 800 mg (1-1-1) sowie Lamotrigin 25 mg (1-1-2) als Off-label-Einsatz zur Phantomschmerzbehandlung. Zusätzlich nahm der Patient Ramipril Hexal 50 mg (1-0-0) sowie Amlodipin 5 mg (1-0-0) zur Blutdrucksenkung ein. Unter der Medikation spürt er kaum Schmerzen und kann sich gut auf seine Aufgaben konzentrieren. Wenn die Medikamente in ihrer Wirkung nachlassen, kommt es zu einer Steigerung der Schmerzen bis zu einem Wert von 7. Er berichtet, sich wegen des fehlenden Arms und der anschließenden Beeinträchtigungen oft niedergeschlagen, antriebsgemindert und „ausgebremst“ zu fühlen. Oft ist er müde und nichts macht ihm Freude. Auch frühere Sozialkontakte finden kaum noch statt. Nach dem Unfall musste er eine Umschulung machen, die mit einer Reduzierung seiner vorherigen Einsatzmöglichkeiten verbunden war und wenig zufriedenstellend für ihn ist. Zur diagnostischen Abklärung psychischer Störungen wurde ein Strukturiertes Klinisches Interview für DSM-IV (SKID‑I und SKID-II; [5]) durchgeführt. Im SKID‑I waren die Kriterien für eine aktuelle depressive Episode erfüllt. Der SKID-II war ohne Befund. Auch das Screening für Somatoforme Störungen (SOMS‑2; [3]) nach ICD-10 und DSM-IV blieb ohne Befund.

## Diagnosen

- Phantomschmerz der linken oberen Extremität mit Teleskop-Phänomen (ICD-10: G54.6)
- Psychologische Faktoren oder Verhaltensfaktoren bei andernorts klassifizierten Krankheiten (ICD-10: F54)
- Mittelgradige depressive Episode (ICD-10: F32.1).

Als Therapieziel formulierte der Patient die Reduktion der Phantomschmerzen ohne Medikation. Um das für die Behandlung von Phantomschmerz etablierte Spiegeltraining zu verwenden, wurde zunächst eine Reduktion des Teleskop-Phänomens angestrebt. Das Teleskop-Phänomen beschreibt eine veränderte (meist verkürzte) Körperrepräsentation des Phantomglieds, die häufig mit verstärkten Phantomschmerzen einhergeht [4]. Es wurde gezeigt, dass Personen mit einem teleskopierten Phantom weniger von der Spiegeltherapie profitieren [2], was mit der diskrepanten Wahrnehmung des Arms im Spiegel und des wahrgenommenen Phantomarms zusammenhängen dürfte. Diese diskrepante Wahrnehmung könnte auch die Kontrolle über das Phantom („sense of agency“) beeinträchtigen, die ebenfalls den Erfolg der Spiegeltherapie vorhersagt [2]. Deshalb sollte die Diskrepanz des visuellen Feedbacks zwischen gespiegeltem Arm und dem verkürzten Phantomarm im Vorwege maximal reduziert werden.

Es wurden 57 Sitzungen kognitiver Verhaltenstherapie von September 2020 bis Dezember 2021 durchgeführt. Nach Erarbeitung eines neuropsychologischen Erklärungsmodells für die Phantomschmerzen [1] wurden sensorisch-perzeptive Empfindungen mittels verschiedener Stimulationsinstrumente aus der quantitativen sensorischer Testung provoziert (eine klassische Bestimmung von Schmerzschwelle, „wind-up“, Allodynie o. Ä. fand nicht statt). Der Pinprick-Stimulator (256 mN), appliziert unter der Achsel an Bizeps und Trizeps, erwies sich als wirksamer Reiz und provozierte ein Kribbeln in der Phantomhand. Mithilfe geleiteter Visualisierungen zur Veränderung des Teleskops gelang es dem Patienten sich vorzustellen, wie er seine Hand wie einen Luftballon aufbläst, während die Therapeutin einen Luftballon aufpustete. Die identifizierten Veränderungsreize wurden in Form von je zwei Imaginationsübungen (Luftballon aufblasen, ein Fernglas herausziehen) und Stimulationseinheiten (taktile Stimulation der Achsel mithilfe einer stumpfen Nadel sowie Drücken mit der Hand unter der Achsel und am Schulterblatt) in die tägliche Routine des Patienten integriert. Die Imaginationsübungen erweiterte der Patient eigenständig um Pumpbewegungen und das Spreizen der Phantomhand. Der leichte Druck an der Achsel führte zu einem Kribbeln im Phantom, welches der Patient mit dem Anstoßen eines Ellenbogens an einer Kante verglich. Die Erweiterung mit gleichzeitigem Drücken unter der Achsel und am Schulterblatt führte zu einem stärkeren Kribbeln in der gesamten Phantomhand. Der Patient berichtete bereits zwei Wochen nach der Einführung der Übungen, eine Verlängerung des Phantomunterarms sowie dessen Beweglichkeit wahrzunehmen.

Nach 6 Wochen wurde der Patient zum selbstständigen Trainieren mit einer Spiegelbrille angeleitet (Hardware: 3‑D-Brille, Elegiant Ltd., www.elegiants.com, verbunden mit Samsung Galaxy Note 4 Smartphone, Model SM-N910F; Software: bootcamp, Android 6.0.1), die es mit einem Weitwinkelobjektiv ermöglichte, seinen gesunden Arm im Raum zu spiegeln. Dabei wurde das Spiegeltraining an das Teleskop-Phänomen angepasst: Der Patient sollte zunächst seine gespiegelte gesunde Hand in genau die gleiche verkürzte Position der Phantomhand bringen und sich auf einer NRS von 0 bis 10 fragen, wie stark das Bild seinem Phantomglied entsprechen würde. Wenn die Abfrage einen Wert von mindestens 9 ergab, so sollte der Patient seinen gesunden Arm ein minimales Stück nach vorne positionieren und sich die gleiche Frage erneut stellen. Ergab diese Abfrage ein Ergebnis von 7 bis 8, konnte mit der Übung begonnen werden; bei kleineren Werten sollte der gesunde Arm wieder etwas mehr nach hinten gezogen werden. Erste Übungen umfassten das Greifen nach einer Tasse sowie das Kneten eines Gummiballs. Der Patient wurde angewiesen, jeden Tag 15 min Imaginationstraining zur Beeinflussung seines Teleskop-Phänomens und 15 min Spiegeltraining durchzuführen. Er implementierte diese zwei Trainingseinheiten in seinen Alltag und führte das Training mit immer wieder individuell an seine Beweglichkeit angepassten Übungen durch. Bereits nach zwei Wochen berichtete der Patient, seine Medikation, vor allem zur Mittagszeit, zu vergessen, da er keinen Anstieg der Schmerzen erlebe. Außerdem merke er keine „Wetterfühligkeit“ mehr. Die Beweglichkeit des Phantomarms zeigte sich ebenfalls erweitert; seitliches Heben sowie eine vertikale und laterale Rotation des Arms seien zu diesem Zeitpunkt bereits möglich. Bei einer Verlaufsmessung 3 Monate nach Einführung des Imaginationstrainings war eine deutliche Verlängerung seines Teleskops von ehemals 9 cm Abweichung zur intakten Hand auf nur noch 3 cm zu verzeichnen.

Es wurde zunächst in Kooperation mit dem Hausarzt die Schmerzmedikation mit Lamotrigin reduziert. Mithilfe therapeutischer Motivationsstrategien wurde der Patient ca. 3 Monate nach Beginn des Spiegeltrainings ermutigt, einen Termin bei einer Schmerzmedizinerin zu vereinbaren, unter deren Monitoring er zunächst Gabapentin über eine Dauer von 4 Monaten und dann im Anschluss innerhalb von 5 Wochen Morphin komplett reduzierte (jeweils im Abstand von 2 bis 3 Wochen eine Dosis am Tag ausgelassen).

Der Patient begann im Frühjahr 2021, die Imaginationsübungen während regelmäßiger Spaziergänge durchzuführen und die Position des linken und rechten Arms sowie deren Schwingbewegungen zu vergleichen. Infolgedessen gelang es ihm, den Phantomarm als natürliche Bewegung weiter nach hinten zu schwenken. In die freiere Gestaltung des Spiegeltrainings wurden außerdem leichte Alltagsaktivitäten, wie ein Kabel drehen, eingebaut.

Durch die Steigerung seines Aktivitätsniveaus stabilisierte der Patient sein Herz-Kreislauf-System, nahm ca. 10 kg ab und benötigte keine Blutdrucksenker mehr. Die restlichen Sitzungen widmeten sich der Zurückeroberung seines alten bzw. eines neuen Lebens. Zur Erhaltung der Schmerzfreiheit nach der erfolgreichen Behandlung der Phantomschmerzen wurde der Patient angewiesen, 2‑ bis 3‑mal pro Woche während seiner Spaziergänge seinen Phantomarm gezielt anzusteuern und die Beweglichkeit zu testen.

Einschränkungen: Es handelt sich um eine unkontrollierte Einzelfallstudie. Placeboeffekte wurden nicht getestet.

## Fazit für die Praxis

- Imaginationsübungen sowie taktile Stimulation halfen, das Teleskop-Phänomen zu reduzieren.
- Ein an die Phantomwahrnehmung (in Position, Lage und Schnelligkeit der Bewegungsabläufe) angepasstes Spiegeltraining führte zu einer willentlich beeinflussbaren Mobilisierung des Phantomglieds („sense of agency“).
- Spiegeltraining reduzierte den Phantomschmerz.
- Unspezifische Effekte wurden nicht kontrolliert.
